# Recognizing Manual Activities Using Wearable Inertial Measurement Units: Clinical Application for Outcome Measurement

**DOI:** 10.3390/s21093245

**Published:** 2021-05-07

**Authors:** Ghady El Khoury, Massimo Penta, Olivier Barbier, Xavier Libouton, Jean-Louis Thonnard, Philippe Lefèvre

**Affiliations:** 1 Service d’Orthopédie et Traumatologie, Cliniques Universitaires Saint-Luc, 1200 Brussels, Belgium; Olivier.barbier@uclouvain.be (O.B.); xavier.libouton@uclouvain.be (X.L.); 2 Institue of Neurosciences (IoNS), Université catholique de Louvain, Avenue Mounier 53, 1200 Brussels, Belgium; massimo@arsalis.com (M.P.); jean-louis.thonnard@uclouvain.be (J.-L.T.); philippe.lefevre@uclouvain.be (P.L.); 3 Arsalis SPRL, Chemin du Moulin Delay 6, B-1473 Glabais, Belgium; 4 Institute of Information and Communication Technologies, Electronics and Applied Mathematics (ICTEAM), Université Catholique de Louvain, 1348 Louvain-La-Neuve, Belgium

**Keywords:** manual activities, activity recognition, movement classification, remote health monitoring, outcome assessment, questionnaires, clinical evaluation

## Abstract

The ability to monitor activities of daily living in the natural environments of patients could become a valuable tool for various clinical applications. In this paper, we show that a simple algorithm is capable of classifying manual activities of daily living (ADL) into categories using data from wrist- and finger-worn sensors. Six participants without pathology of the upper limb performed 14 ADL. Gyroscope signals were used to analyze the angular velocity pattern for each activity. The elaboration of the algorithm was based on the examination of the activity at the different levels (hand, fingers and wrist) and the relationship between them for the duration of the activity. A leave-one-out cross-validation was used to validate our algorithm. The algorithm allowed the classification of manual activities into five different categories through three consecutive steps, based on hands ratio (i.e., activity of one or both hands) and fingers-to-wrist ratio (i.e., finger movement independently of the wrist). On average, the algorithm made the correct classification in 87.4% of cases. The proposed algorithm has a high overall accuracy, yet its computational complexity is very low as it involves only averages and ratios.

## 1. Introduction

Hands can be affected in different neurologic, rheumatologic, degenerative or traumatic conditions. To evaluate this manual impairment, physicians rely on medical history and clinical examination, but have also several tools at their disposal. For instance, they can use diagnostic tests such as electromyography and patient-reported outcome measures that reflect the patient’s point of view [1]. Motion capture analysis can also provide additional information, though it is more commonly used in research rather than in a routine clinical setting. Medical practice has shifted towards evidence-based treatments with the aim of providing the best results when treating patients. Therefore, robust outcome evaluations are needed to assess the effectiveness and reliability of a treatment [2]. 

An activity is defined in the International Classification of Functioning, Disability and Health (ICF) as the execution of a task or action by an individual [3]. Measuring the activity domain is a key point in determining the impact of different treatments on functional recovery, as the consequences of a pathology on patients’ functioning are the most manifest through their inability to carry out activities of daily living (ADL) [4]. Activity performance cannot be measured directly, but can either be inferred by direct observation, which is time consuming in practice, or can be self-reported by patients through questionnaires.

Questionnaires can provide self-reported measures focused on the patients’ perceptions of their activity limitations. They inform clinicians on how well patients manage their activity in their home environment. For example, ABILHAND is a questionnaire that measures manual ability through activities that present a common perceived difficulty among patients [5]. It provides an invariant linear scale allowing quantitative comparisons of manual ability between patients and over time. The units of this scale are expressed in logits, and can be converted into centiles for a more intuitive clinical interpretation. The scale has been validated in populations with various pathologies [6,7,8,9,10,11]. Other questionnaires such as the Disabilities of the Arm, Shoulder and Hand (DASH) [12], the Patient-Rated Wrist Evaluation (PRWE) [13] and the Carpal Tunnel Questionnaire (CTQ) [14] have been developed to measure different aspects of upper limb function. These self-reported measures are based on the respondent’s memory of the perceived difficulty and their ability to accurately judge their capability [15]. Items that compose these questionnaires are representative of the patients’ daily manual activities (e.g., using a spoon or tying shoelaces).

Another complementary approach to that of the questionnaires would be a direct assessment of the patient’s actual activities. A direct assessment could be used to monitor a patient’s actual activity objectively, without relying on the patient’s memory, and systematically, witnessing what activities the patient actually does or does not do. The ability to monitor activities of daily living in the patient’s natural environment could become a valuable tool for clinical decision-making, evaluating healthcare interventions, and supporting and tracking rehabilitation progress. Inertial sensors have been used for monitoring activities as they are small, affordable, and generally unobtrusive [16]. They have been used for upper limb motion analysis with good accuracy and reliability [17,18]. They have been shown to be useful for clinical applications [19], and proved to be more sensitive than questionnaires to detect changes in shoulder movement, thus adding a complementary objective component to outcome measurement [20].

Different authors have worked on recognizing upper limb movements using accelerometry alone [21,22] or in combination with surface electromyography [23], and on building devices that could track hand use [24]. For instance, the “manumeter” determines hand use by tracking the total angular distance traveled by the wrist and fingers using magnetometers [24]. This device is able to track global hand use, but performs poorly for tasks requiring small yet intensive movements such as handwriting [25]. Another limitation is the interaction with ferromagnetic objects, which are commonly used in everyday life, and can alter the device readings.

As a complementary approach to questionnaires that are common to a patient population, a hand activity monitoring device that not only tracks the global hand use, but is also able to categorize the manual activities that are actually performed, would offer a more personalized approach and would have implications in many aspects of patient care. In this paper, we show that a simple algorithm is capable of classifying manual activities of daily living using data from wrist- and finger-worn sensors. 

## 2. Materials and Methods

### 2.1. Prototype

**Figure 1:**
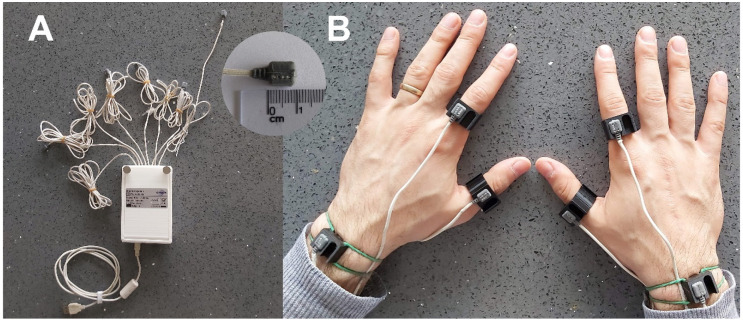
(**A**). Photograph showing the device prototype, which consists of eight inertial measurement units connected to a processor. A close-up of one of the sensors is shown. (**B**). Photograph showing the placement of the sensors on 3D-printed supports on the participant’s hands.

We used a prototype device (InSense©, Arsalis, Belgium) to capture human activity signals using inertial measurement units (IMUs). The device is shown in Figure 1A. Each sensor integrates a triaxial accelerometer and a triaxial gyroscope. The measurement range is ±16 g and ±2000°/s for each axis of accelerometer and gyroscope, respectively. The device is wired and transmits sampled sensor data to a laptop computer via a USB interface. The sensors are small in size (9.4 × 8 × 5.5 mm) and lightweight enough (2 g) to be worn comfortably without altering the hand movements. The inertial signals of all sensors are sampled synchronously (inter sensor delay < 0.125 ms) with a 16-bit resolution at a rate of 500 Hz.

### 2.2. Sensor Calibration

Accelerometers and gyroscopes were calibrated prior to performing the experiments so that the readings were accurate and reliable. Accelerometers were calibrated by applying 0 g, 1 g and −1 g on each accelerometer of each sensor. Their calibration reported an average absolute error of 0.18% of full scale (FS) on any axis of any sensor (range: 0.05 to 0.62 %FS). Gyroscopes were calibrated using a rotating device equipped with a 1024 point resolution optical encoder that was used to determine the reference angular speed (Video S1 in Supplementary Materials). They were calibrated at angular speeds ranging from −600 to +600°/s and reported an average absolute error of 0.23 %FS on any axis of any sensor (range: 0.13 to 0.71 %FS). Raw data were converted to physical values of angular velocity and acceleration expressed in °/s and g, respectively, using individual sensor calibration coefficients.

### 2.3. Participants

Six healthy adults participated in this study; their characteristics are detailed in Table 1. Participants were included in the study if they were above 18 years old and had no pathology that could affect the use of their upper limbs. The study was conducted according to the guidelines of the Declaration of Helsinki, and approved by the ethics committee of Cliniques Universitaires Saint-Luc Université catholique de Louvain (2015/26JAN/025, N° B403201523492). Participants provided written informed consent to make use of their anonymized data.

### 2.4. Activities Selection

In order to explore the wide range of hand movements, activities were selected from the different pathology-specific versions of the ABILHAND questionnaire (hand surgery, stroke and rheumatoid arthritis). Items from this questionnaire have been rigorously selected to report patient-perceived difficulty unbiased by patient demographics (e.g., age, gender) nor clinical conditions (e.g., side affected, manual ability). Twelve activities were selected to cover the whole range of measurement of the ABILHAND scale. Two additional items were added for their relevance in everyday life, namely “typing on a computer keyboard” and “using a spoon”. The final list of 14 activities is shown in Table 2. We hypothesized that these activities could be classified into five different categories, based on the way they are actually executed. Some activities are unimanual while others are bimanual. Bimanual activities could require the action of a stabilizing hand or involve both hands equally. In addition, some activities require the use of the fingers (the fingers move independently of the wrist), while others involve the whole hand (the fingers move together with the wrist), for example, when manipulating a tool. When some manual activities could be performed in different ways (e.g., some participants brushed their hair with both hands while others used only their dominant hand), the experimenter’s judgement was used to classify each activity into a category, depending on the way it was executed by the participant.

### 2.5. Experimental Setup and Recordings

Participants were equipped with the prototype device sensors on the first phalanges of the first two fingers of both hands and on the wrists (Figure 1B). Sensors were fitted on 3D-printed supports in the shape of rings for the fingers and wristbands for the wrists. These sites were chosen to correspond to sites where everyday accessories are worn (watch and rings) and do not hinder activities of daily living.

Participants were asked to perform the 14 activities in a random order for five repetitions each, while sitting on a chair at a table. The tools used (e.g., can opener, pen) were from the participants’ home environment. They were instructed to perform each activity as they would do in their normal life, with no constraints except for the duration of each activity (no more than 25 s per repetition). Each activity started and ended with the hands still on the table, separated by five seconds of inactivity. Experiments were performed under the supervision of the experimenter.

### 2.6. Data Analysis

Each recording was processed to isolate the activity period (i.e., when the participant is actually executing the task) from inactivity periods (between two consecutive repetitions). The main goal was to focus on activity recognition, based on the assumption that the start and end of an activity were known.

Gyroscope signals were used to analyze the angular velocity pattern for each activity, as they demonstrated the most distinctive pattern compared to the accelerometers. No filter was applied to the raw data. For each gyroscope signal, the norm of the angular velocity vector was computed by combining the x, y and z components. Signals were combined to compute the hand signal (mean of the three IMUs on one hand) and the fingers signal (mean of the two IMUs placed on the fingers) for both limbs. The elaboration of the algorithm was based on the examination of the activity at the different levels (hand, fingers and wrist) and the relationship between them for the duration of the activity.

The hands ratio (HR) was calculated by dividing the angular velocity of the most active hand by that of the least active one.

(1) $$HR=\frac{Most\;active\;hand}{Least\;active\;hand}$$

It was chosen as a criterion to differentiate between bimanual activities involving both hands equally and those involving a stabilizing hand. When both hands are involved equally, the HR is expected to be close to one. During unimanual activities or when one hand stabilizes an object, one hand is less active than the hand performing the movement, and the HR is expected to increase.

The fingers-to-wrist ratio (FWR) was computed by dividing the fingers’ angular velocity (mean of both fingers) by the wrist angular velocity.

(2) $$FWR=\frac{Fingers’\;angular\;velocity}{Wrist\;angular\;velocity}$$

When the hand moves as a whole (e.g., when manipulating a hammer), the angular velocity in the fingers is close to that of the wrist; hence, the FWR is close to one. If the fingers are involved in independent movements (e.g., when writing), the FWR increases. The FWR was computed on the dominant hand for bimanual activities with a stabilizing hand, and by taking the average of the two hands for bimanual activities.

### 2.7. Determining Cutoff Points

The Receiver Operating Characteristic (ROC) curve was used to determine the cutoff points for HR and FWR that best discriminate between the different categories of activities [26]. The ROC curve is a graphical plot that illustrates the diagnostic ability of a binary classifier system as its discrimination threshold is varied. The ROC curve plots sensitivity (i.e., the true positive rate) against 1–specificity (i.e., the false positive rate) at various threshold settings. The optimal cutoff value (i.e., threshold) was chosen as the point that jointly maximized sensitivity and specificity, hence leading to the least number of misclassifications. The area under the curve (AUC) is the measure of the ability of the classifier to distinguish between classes. The greater the AUC, the better the criterion is able to distinguish between the different categories. AUC values between 0.7 and 0.8 are considered acceptable, and values above 0.8 are considered to have excellent discrimination levels [27]. 

### 2.8. Algorithm Validation

**Figure 2:**
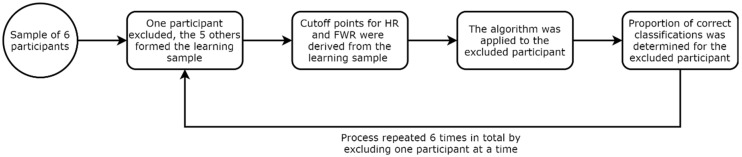
Diagram showing the validation process of the algorithm.

A leave-one-out cross-validation was used to validate our algorithm [28], as detailed in Figure 2. For each iteration of the validation, one participant was left out of the training sample, and data from the five remaining participants were used to derive cut-off values for the HR and FWR and establish the algorithm. The latter was then applied to the data of the participant left out to evaluate the performance of the algorithm. Each individual repetition was categorized using these cutoffs and following the steps laid by the algorithm. This process was repeated six times in total to compute the validation errors. Activities were considered correctly identified into their respective categories if both criteria (HR and FWR) for this category were in the right range at each step of the algorithm. The performance of the algorithm was then calculated by comparing the activity category as established by the experimenter and the categorization provided by the algorithm.

## 3. Results

### 3.1. Cutoff Points

**Figure 3:**
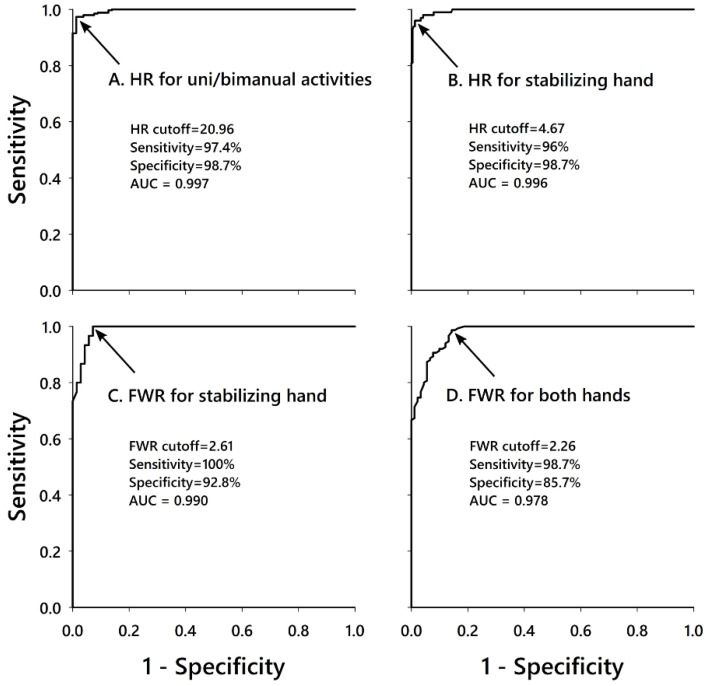
Receiver-operating-characteristic curve showing the cut-off points for the hands ratio (HR) and the fingers-to-wrist ratio (FWR). The arrows show the point that maximizes sensitivity and specificity. (**A**) HR for the discrimination between uni- and bimanual activities. (**B**) HR for the discrimination between activities involving a stabilizing hand and those involving both hands. (**C**) FWR for identifying finger activity in activities involving a stabilizing hand. (**D**) FWR for identifying finger activity in activities involving both hands. AUC: Area Under the Curve.

The ROC curves used to determine the cutoff points for the hands ratio (HR) and fingers-to-wrist ratio (FWR) for the whole sample are shown in Figure 3. The sensitivity ranged between 96% and 100%, and the specificity from 85.7% to 98.7%. The AUCs for all criteria were above 0.978, providing excellent discrimination. The individual values for the different iterations of the leave-one-out cross-validation are detailed in Table 3. These did not vary substantially in comparison with values for the whole sample, demonstrating the robustness of the approach.

### 3.2. Description of the Algorithm

**Figure 4:**
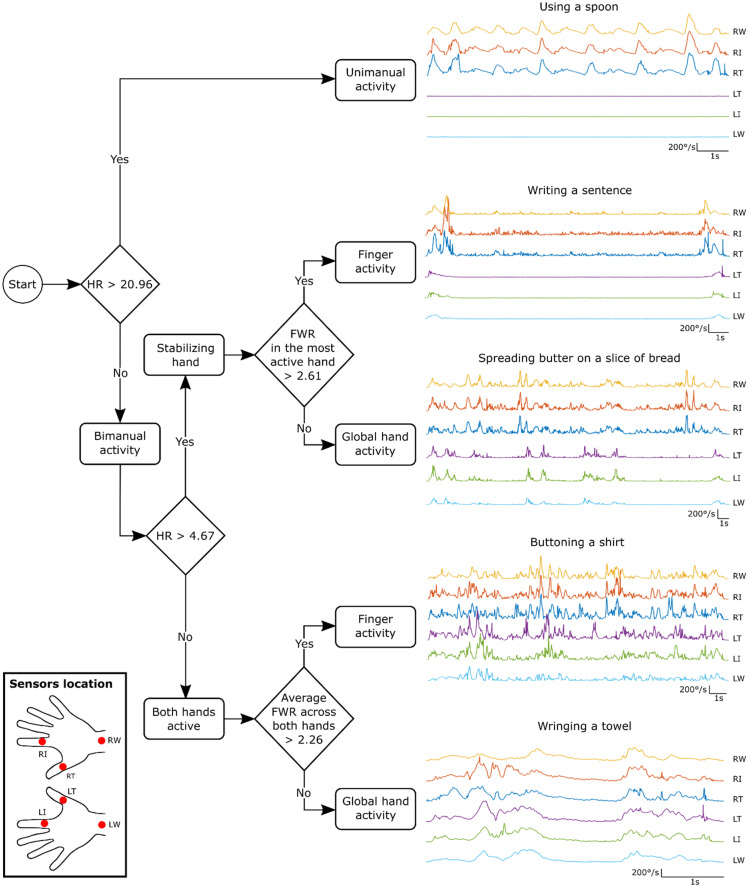
Algorithm for the classification of manual activities. The values shown for the hands ratio (HR) and fingers-to-wrist ratio (FWR) are those extracted from the whole sample. Typical traces for five tasks performed by a right-handed subject show the signals of the six sensors for one repetition of one selected activity for each category. RW: Right Wrist, RI: Right Index; RT: Right Thumb; LT: Left Thumb, LI: Left Index; LW: Left Wrist.

The algorithm (Figure 4) allows the classification of manual activities into five different categories through three different steps, based on HR and FWR. The first step of the algorithm separates unimanual from bimanual activities based on HR. A HR greater than 20.96 is indicative of unimanual activities, i.e., one of the hands is over 20 times more active than the other hand. For the second step of the algorithm, a cutoff HR of 4.67 can be used to separate bimanual activities that use a stabilizing hand from those that involve both hands equally. The HR for this second step is smaller than that of the first step, as the stabilizing hand still performs low amplitude movements. The third step separates activities based on whether the movement involves the fingers or not. A FWR larger than 2 (actually 2.61 for bimanual activities involving a stabilizing hand and 2.26 for bimanual activities involving both hands equally) means that the fingers are about two times more active than the wrists, indicating that the fingers are mainly performing the movement such as when writing or buttoning a shirt. On the contrary, “spreading butter on a slice of bread”, for example, involves using the hand as a whole when manipulating a tool (global hand activity, FWR < 2). In summary, the algorithm can classify manual activities based on the involvement of the hands relative to one another, and the presence or absence of finger activity.

### 3.3. Performance of the Algorithm

Cutoff values for HR and FWR were derived from the learning sample and then tested for validation on the participant left out using the classification algorithm. An example of the validation method is shown in Table 4. For each repetition of each activity, the HR and FWR were extracted for participant 3. Each one of these values was then compared to the cutoffs derived when excluding participant 3 (see Table 3), according to the steps previously detailed in the algorithm. When the observed value was in the expected range, the cell was colored in green. When outside the range, it was colored in red. For example, the HR for the first repetition of “Using a spoon” was 56.73, which is >20.96 and, thus, verified the criteria for being a unimanual activity. The FWR for the fifth repetition of “Opening a screw-topped jar” was 2.43, which was slightly above the expected value for a bimanual activity with global activity of both hands (the FWR should be <2.26). The observed value was outside the range, and the cell was colored in red. This process was repeated for each one of the six participants, and the sum of correct classifications was computed.

The performance of the algorithm on the validation sample is detailed in Table 5. Each column in the table represents a step in the algorithm, and the percentage of correct classification is detailed for the classification of each activity in the correct category.

For the first step, the algorithm was able to classify uni- from bimanual activities based on HR with an average accuracy of 97%. The activity “writing a sentence” was incorrectly classified in 33% of the cases as a unimanual activity. This is explained by the fact that the stabilizing hand is only active at the beginning and the end of the movement, and thus, has little influence on HR, especially as the activity lasts longer. This misclassification originated almost exclusively from two subjects (nine out of ten incorrect classifications).

For the second step of the algorithm, activities requiring a stabilizing hand were classified correctly in 95% of cases and those involving both hands equally in 98% of cases. The third step correctly identified the presence or the absence of fingers’ involvement in 89 to 100% of cases, per category. 

For an activity to be classified in the correct category, it had to verify the HR and FWR criteria for every repetition. On average, this was achieved in 87.4% of the activities, as shown by the overall accuracy in the last column of Table 5. 

## 4. Discussion

In this paper, we show the applicability of a very simple algorithm for the categorization of 14 manual ADL. Using gyroscope data from six IMUs located on the thumb, index finger and wrist of both hands, we were able to classify manual ADL into five categories. The proposed algorithm has a high overall accuracy, yet its computational complexity is very low as it involves only averages and ratios of sensor measurements.

Our algorithm was able to classify manual activities into their correct category in 87.4% of cases. The poorest performance in categorization corresponded to the activity “writing a sentence” (category “bimanual activities with a stabilizing hand and finger activity of the active hand”), for which the accuracy was 67% on average. Our results show that it is a borderline activity that can be performed using only one hand if the support is stable enough. Contrary to lower limb movements, most manual activities are complex to analyze, mainly because they are non-cyclical and variable [29]. Differences in movement patterns exist across individuals and across repetitions by the same individual. This was especially evident in our study for the activity “brushing one’s hair”. Participants used either one or both hands to brush their hair, and the activity was, thus, considered either unimanual or bimanual depending on the actual performance. In practice, this misclassification can be tolerated and only highlights the variability across all subjects and movement patterns used to perform these manual ADL. Nevertheless, most activities performed in this study were conducted in a similar manner across subjects and repetitions, which is encouraging for the future automated applications of the algorithm.

Classifying activities into different categories is an important first step, because manual activities that belong to the same category are likely to be equally impaired in a given pathology since they involve the same movement pattern. Indeed, the perceived difficulty of the activities of ABILHAND has shown that, for instance, for stroke patients, manual activities are more challenging if they require both hands and even more challenging if they involve the fingers of both hands [7]. In rheumatoid arthritis, challenging activities are those that involve higher stress at the joints, whether uni- or bi-manual [6]. Therefore, for clinical follow-up of manual activity, we can hypothesize that the achievement of a type of activity is likely a very good indicator of recovery. In addition, some activities are usually only seldom performed during the day (e.g., “tying shoelaces” and “buttoning a shirt”), and grouping them as categories allows continuous monitoring whatever actual activities are performed during the day. Another argument for grouping the activities is the ability to target patients with different occupational profiles. For example, an office worker would spend most of the day typing on a keyboard or writing, while a manual worker would, rather, manipulate tools.

One strength of our study is the selection of activities that have been shown to characterize manual ability in patients with various pathologies [6,7,10]. The possibility to recognize the activity categories, or, in a later step, the execution of these individual activities in daily life will pave the way for comparisons between the patient-reported questionnaire scores and objective automated monitoring. Indeed, the correlations observed between the kinematic analysis of the upper limb, questionnaire scores and observational methods [30,31] indicate that an approach combining objective activity monitoring and questionnaire scores could help clinicians in the selection of the optimal treatments for their patients. Using such a combined approach, clinicians will better discern between capability, which describes what the patient can do in their daily environment, from performance, which refers to what the patient actually does [15]. 

The upper extremity is conceptualized as a single functional unit with the shoulder, elbow and wrist joints used to position the end-effector organ, the hand, in space. The chosen localizations for the sensors allowed the capturing of the functioning of both hands very well. The wrist sensors are able to measure the movement of the hand in space, while the finger sensors record the movement of the fingers. The presence of sensors on the thumb and index finger allowed our device to be sensitive to movements of the hand involving different types of pinches and grasps (e.g., writing and handling tools), as well as activities involving fine finger movements (e.g., typing) [32]. The addition of sensors on other locations, such as the third finger and the fingernails for precise manipulation, and the fifth finger for power grasping, could possibly provide more information regarding the type of movement. However, this additional information would come at the cost of obtrusiveness and a plethora of data. The number of sensors used in the current study is higher than in similar studies dealing with recognizing activities of the upper limb [21,22]. However, they provide a very good amount of data for the development of a more complex algorithm, and their location corresponds to that of everyday accessories (rings and wristbands), allowing the definitive monitoring device to be unobtrusive and ergonomic.

Cut-off values were found to be quite similar across the different analyses, except for that of the fourth participant, whose exclusion yielded slightly different results. The stability of the HR and FWR is promising regarding the generalization of the algorithm to a larger population. Participants performed the ADL as they would do in their normal life and with objects of their home environment. Unconstraining the experiment in this manner helped to generate a wide range of variability in the data, which could ultimately result in the development of an algorithm that is more readily applicable in real life. We obtained very good results in spite of potential measurement errors due to the small displacement of the sensor over the skin. 

Commonly used pattern recognition approaches are neural networks, structural matching, template matching and statistical classification [33,34]. The latter approach was used in the present paper, in which each pattern is represented in terms of features of measurement. This has proven effective in developing a simple algorithm for hand activities’ classification. Results are encouraging and show that activities can be reliably detected in normal subjects performing unconstrained movements. Future research should include a larger sample size to test for the stability of our chosen cutoffs, and testing of the algorithm on patients with an impaired hand function. Improvements in the algorithm could be made by using artificial intelligence (e.g., machine learning and pattern recognition), which could ultimately distinguish between individual activities. With these improvements, one should be able to determine the benefit of recognizing individual activities compared to categories. The simpler process of categorizing activities might prove sufficient for clinical applications. Nonetheless, substantial impairments can alter the execution of an activity through compensatory mechanisms, and more sophisticated algorithms might prove more appropriate in this case. A critical development of the current prototype would be an extension to a wireless system connected to a smartphone. This would allow recognition of manual activities as well as the context in which they are carried out (e.g., while sitting or walking). Recognizing the beginning and end of an activity was not addressed in this paper, but will be an essential step for the future implementation of the monitoring device in real life.

Using the monitoring device in combination with the questionnaires, the clinician will be able to optimize the patient’s treatment and follow-up. A clinical improvement should manifest into more hand use and, thus, more ADL recorded on the device, as well as higher scores on the questionnaires due to a decrease in perceived difficulty. The physician will also be able to personalize the patient’s therapy by tracking and focusing on a particular activity that is judged as important for the patient. 

Ultimately, we aim at developing a manual activity monitoring device with wireless sensors and an autonomous power supply in order to capture manual activities in the patient’s natural environment. The compatibility of the chosen locations for the sensors with everyday life accessories will not hinder the execution of ADL. Gathering objective data from this device could be combined with patient-reported data from questionnaires in order to provide a comprehensive and global approach for outcome evaluation, clinical decision-making, patient monitoring and the tracking of rehabilitation progress. 

## Figures and Tables

**Table 1:** Participants’ characteristics.

	Age	Sex	Height (cm)	Weight (kg)	Work
Participant 1	31	F	152	42	Office worker
Participant 2	65	M	162	80	Dentist
Participant 3	28	M	173	74	Office worker
Participant 4	24	F	176	78	Student
Participant 5	31	M	171	70	Office worker
Participant 6	57	F	164	53	Housewife

**Table 2:** List of manual activities and their respective categories.

	Activity	Category
1	Using a spoon	Unimanual
2	Drinking a cup of water	
3	Brushing one’s hair	
4	Writing a sentence	Bimanual with a stabilizing hand and finger activity of the active hand
5	Spreading butter on a slice of bread	Bimanual with a stabilizing hand and global activity of the active hand
6	Opening a can with a can opener	
7	Typing on a computer keyboard	Bimanual with finger activity of both hands
8	Shuffling and dealing cards	
9	Peeling potatoes with a knife	
10	Buttoning a shirt	
11	Tying shoelaces	
12	Opening a screw-topped jar	Bimanual with a global activity of both hands
13	Lifting a full pan	
14	Wringing a towel	

**Table 3:** Cut-off values for the hands ratio and the fingers-to-wrist ratio.

	HR ^1^ for Classification between Uni- and Bimanual Activities	HR ^1^ for Classification between Bimanual Activities Involving a Stabilizing Hand and Those Using both Hands	FWR ^1^ for Fingers Involvement of Bimanual Activities Using a Stabilizing Hand	FWR ^1^ for Fingers Involvement of Bimanual Activities Using both Hands
Participant 1 excluded	20.96	4.25	2.68	2.50
Participant 2 excluded	20.96	4.67	2.61	2.42
Participant 3 excluded	20.96	4.67	2.61	2.26
Participant 4 excluded	22.01	4.71	2.56	2.26
Participant 5 excluded	20.96	4.67	2.61	2.42
Participant 6 excluded	20.95	4.62	2.61	2.25
Whole sample	20.96	4.67	2.61	2.26

^1^ HR: Hands Ratio; FWR: Fingers-to-Wrist Ratio.

**Table 4:** Validation of the algorithm for participant 3 ^1^.

					Step 1						Step 2						Step 3				
					HR ^2^ for Uni/Bimanual Activities						HR ^2^ for Stabilizing Hand/both Hands Active						FWR ^2^ for Finger Activity/Global Hand Activity				
				Cutoff	Rep1 ^2^	Rep2	Rep3	Rep4	Rep 5	Cutoff	Rep1	Rep2	Rep3	Rep4	Rep 5	Cutoff	Rep1	Rep2	Rep3	Rep4	Rep 5
Unimanual activities			Brushing one’s hair	HR > 20.96	32.11	54.26	66.41	68.41	83.61		N/A						N/A				
			Using a spoon		56.73	32.44	41.81	67.44	59.21												
			Drinking a cup of water		48.90	58.22	68.75	68.21	85.08												
Bimanual activities	Stabilizing hand	Finger activity	Writing a sentence	HR < 20.96	27.72	25.11	26.97	20.13	29.52	HR > 4.67	27.72	25.11	26.97	27.54	29.52	FWR > 2.61	2.80	2.72	2.80	2.83	2.68
		Global hand movement	Spreading butter on a slice of bread		7.63	8.10	11.44	10.69	16.35		7.63	8.10	11.44	10.69	16.35	FWR < 2.61	1.72	1.62	1.61	1.61	1.49
			Opening a can with a can opener		5.92	8.52	9.98	11.93	11.41		5.92	8.52	9.98	11.93	11.41		2.29	2.67	2.01	2.10	2.10
	Both hands active	Finger activity	Typing on a computer keyboard		3.21	2.50	2.75	3.11	2.91	HR < 4.67	3.21	2.50	2.75	3.11	2.91	FWR > 2.26	5.77	5.58	5.20	5.29	5.36
			Shuffling and dealing cards		2.80	2.44	2.58	2.59	2.58		2.80	2.44	2.58	2.59	2.58		2.97	3.28	2.93	3.23	3.24
			Peeling potatoes with a knife		1.74	1.97	1.93	1.98	1.94		1.74	1.97	1.93	1.98	1.94		2.80	2.62	2.90	3.07	3.02
			Buttoning a shirt		2.20	2.01	2.42	2.16	2.67		2.20	2.01	2.42	2.16	2.67		3.00	3.13	2.88	3.27	3.16
			Tying shoelaces		2.71	4.27	2.42	3.16	2.61		2.71	4.27	2.42	3.16	2.61		2.75	2.64	2.85	2.60	2.54
		Global hand movement	Opening a screw-topped jar		1.93	1.77	1.78	2.15	1.92		1.93	1.77	1.78	2.15	1.92	FWR < 2.26	2.02	1.98	2.18	1.94	2.43
			Lifting a full pan		1.51	1.52	1.54	1.50	1.51		1.51	1.52	1.54	1.50	1.51		1.70	1.55	1.56	1.50	1.49
			Wringing a towel		2.29	2.17	2.15	2.54	2.38		2.29	2.17	2.15	2.54	2.38		2.01	1.72	2.19	1.87	1.73

^1^ Green and red cells: when the value of the HR or FWR verifies the condition or not, respectively. ^2^ HR Hands Ratio; FWR: Fingers-to-Wrist Ratio; Rep: repetition.

**Table 5:** Performance of the algorithm.

				Step 1 HR ^1^ for Uni/Bimanual Activities		Step 2 HR ^1^ for Stabilizing Hand/both Hands Active		Step 3 FWR ^1^ for Finger Activity/Global Hand Activity		Overall Accuracy	
				Accuracy per Activity	Accuracy per Category	Accuracy per Activity	Accuracy per Category	Accuracy per Activity	Accuracy per Category	Accuracy per Activity	Accuracy per Category
Unimanual activities			Brushing one’s hair (19) ^2^	100%	97%					100%	97%
			Using a spoon	100%		N/A	N/A	N/A	N/A	100%	
			Drinking a cup of water	93%						93%	
Bimanual activities	Stabilizing hand	Finger activity	Writing a sentence	67%	97%	100%	95%	100%	100%	67%	67%
		Global hand movement	Spreading butter on a slice of bread	100%		97%		100%	90%	97%	84%
			Opening a screw-topped jar (10) ^2^	100%		90%		40%		40%	
			Opening a can with a can opener	100%		90%		97%		87%	
	Both hands active	Finger activity	Typing on a computer keyboard	100%		100%	98%	90%	89%	90%	86%
			Shuffling and dealing cards	100%		90%		100%		90%	
			Peeling potatoes with a knife	100%		100%		93%		93%	
			Buttoning a shirt	100%		97%		87%		83%	
			Tying shoelaces	100%		97%		73%		73%	
		Global hand movement	Opening a screw-topped jar (20) ^2^	100%		100%		75%	90%	75%	90%
			Lifting a full pan	100%		100%		100%		100%	
			Wringing a towel	100%		97%		87%		87%	
			Brushing one’s hair (11) ^2^	100%		100%		100%		100%	

^1^ HR Hands Ratio; FWR: Fingers-to-Wrist Ratio ^2^ When participants performed the activity differently, the number between brackets indicates the number of repetitions in the current category.

## Data Availability

The data presented in this study are available in Supplementary Materials 2.

## Supplementary Materials

The following are available online at https://www.mdpi.com/article/10.3390/s21093245/s1, Video S1: Gyroscopes’ calibration procedure.
